# A Large Cohort of Hemoglobin Variants in Thailand: Molecular Epidemiological Study and Diagnostic Consideration

**DOI:** 10.1371/journal.pone.0108365

**Published:** 2014-09-22

**Authors:** Hataichanok Srivorakun, Kritsada Singha, Goonnapa Fucharoen, Kanokwan Sanchaisuriya, Supan Fucharoen

**Affiliations:** 1 Centre for Research and Development of Medical Diagnostic Laboratories, Faculty of Associated Medical Sciences, Khon Kaen University, Khon Kaen, Thailand; 2 Graduate School, Khon Kaen University, Khon Kaen, Thailand; Johns Hopkins Bloomberg School of Public Health, United States of America

## Abstract

**Background:**

Hemoglobin (Hb) variants are structurally inherited changes of globin chains. Accurate diagnoses of these variants are important for planning of appropriate management and genetic counseling. Since no epidemiological study has been conducted before, we have investigated frequencies, molecular and hematological features of Hb variants found in a large cohort of Thai subjects.

**Materials and Methods:**

Study was conducted on 26,013 unrelated subjects, inhabiting in all geographical parts of Thailand over a period of 11 years from January 2002-December 2012. Hb analysis was done on high performance liquid chromatography (HPLC) or capillary electrophoresis (CE). Mutations causing Hb variants were identified using PCR and related techniques.

**Results:**

Among 26,013 subjects investigated, 636 (2.4%) were found to carry Hb variants. Of these 636 subjects, 142 (22.4%) carried α-chain variants with 13 different mutations. The remaining included 451 (70.9%) cases with 16 β-chain variants, 37 (5.8%) cases with Hb Lepore (δβ-hybrid Hb) and 6 (0.9%) cases with a single δ-chain variant. The most common α-globin chain variant was the Hb Q-Thailand (α^74GAC-CAC, Asp-His^) which was found in 101 cases (15.8%). For β-globin chain variants, Hb Hope (β^136GGT-GAT, Gly-Asp^) and Hb Tak (β^146+AC, Ter-Thr^) are the two most common ones, found in 121 (19.0%) and 90 (14.2%) cases, respectively. Seven Hb variants have never been found in Thai population. Hb analysis profiles on HPLC or CE of these variants were illustrated to guide presumptive diagnostics.

**Conclusions:**

Hb variants are common and heterogeneous in Thai population. With varieties of thalassemias and hemoglobinopathies in the population, interactions between them leading to complex syndromes are common and render their diagnoses difficult in routine practices. Knowledge of the spectrum, molecular basis, genotype-phenotype correlation and diagnostic features should prove useful for prevention and control of the diseases in the region.

## Introduction

Hemoglobinopathies are inherited hemoglobin (Hb) disorders. Defects with absent or reduced synthesis of particular globin chains are called thalassemias, while Hb variants or abnormal Hbs are structurally inherited changes of globin chains [1]. Both thalassemia and Hb variants have been increasingly reported worldwide. To date the HbVar database has noted more than 1,150 mutations [2]. While extensive studies have been conducted for both α-thalassemia, β-thalassemias and other globin gene abnormalities in Thailand [3]–[6], less study have been carried out for Hb variants. Although most of the Hb variants are non pathological variants, some could produce clinically relevant phenotypes when found in association with thalassemia or other hemoglobinopathies. Accurate diagnoses of these hemoglobinopathies are important for planning of appropriate management and genetic counseling. However, these are problematic in routine practice and usually require hematological, biochemical and molecular characterizations [7], [8].

Many Hb variants have minimal clinical significance and are sometimes discovered during a systematic study performed within program for prevention of thalassemia or sickle cell disease. In several regions, these are found during a premarital screening or neonatal screening program. With the introduction of several dedicated Hb analyzers which when used in combination could provide reliable identification of many common variants [9], before being confirmed by molecular testing. In Thailand, the average frequency of α-thalassemia is 20–30%, β-thalassemia is 3–9% and that of Hb E is 20–30% [10]. In a micromapping study recently carried out in northeast Thailand, we have identified prevalence of 41.7% for Hb E, 5.8% for α^0^-thalassemia, 31.4% for α^+^-thalassemia and 0.9% for β-thalassemia [11]. Although Hb variants have been sporadically reported in Thai individuals, no information on the prevalence and heterogeneity are available in Thai population. In this study, we have performed a large scale survey of Hb variants in Thai population observed over a period of 11 years in 26,013 subjects living in different geographical areas of the country.

## Materials and Methods

### Subjects and hematological analysis

**Figure 1:**
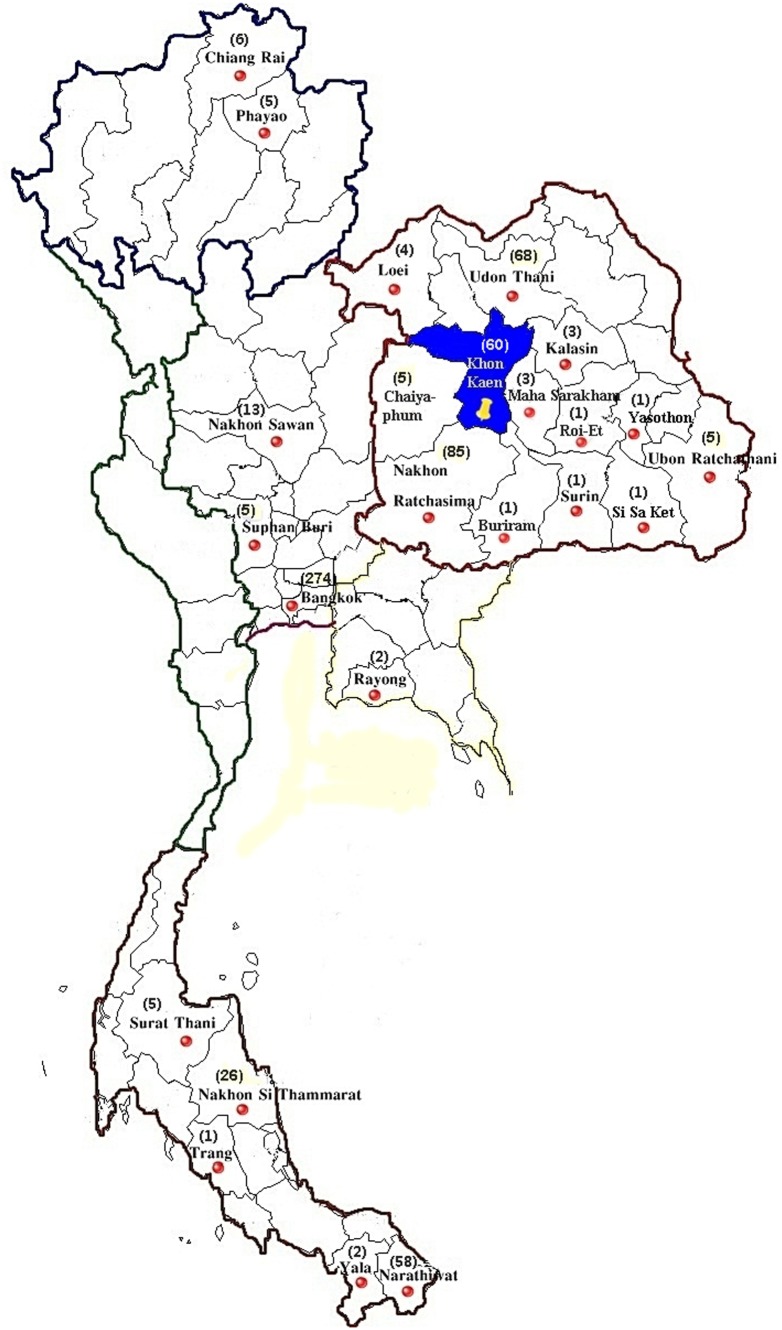
Map of Thailand illustrating 24 provinces in different geographical areas where specimens were recruited at Khon Kaen province in northeast Thailand (highlighted).

Ethical approval of the study protocol was obtained from our Institutional Review Board (IRB) at Khon Kaen University, Thailand (HE510728). Since the study was done on anonymous leftover specimens from routine practice, the IRB of Khon Kaen University waived the need for consent. Retrospective data of known Hb variants were selectively recruited from samples encountered at our thalassemia service centre at Khon Kaen University, Khon Kaen, Thailand, during January 2002 to December 2012. Blood specimens were referred from various hospitals located in many parts of Thailand (**Figure 1**). A total of 26,013 unrelated subjects were reviewed. Hematological and Hb analysis were performed at initial laboratories or at our center where appropriate. Analysis of Hb variants were done using automated capillary zone electrophoresis (Capillarys 2 Flex Piecing: Sebia, Lisses, France) and automated HPLC analyzer (Variant; Bio-Rad Laboratories, Hercules, CA, USA).

### DNA analysis

Genomic DNA was prepared from peripheral blood leukocytes using the standard method. Allele specific PCR assays previously established in our laboratory were used to provide final diagnoses of several known Hb variants including Hb Q-Thailand (α^74GAC-CAC, Asp-His^), Hb Queens (α^34CTGCGG, Leu-Arg^), Hb Siam (α^15GGT-CGT, Gly-Arg^), Hb Hope (β^136GGT-GAT, Gly-Asp^), Hb Phimai (β^72AGT-ACT, Ser-Thr^), Hb Pyrgos (β^83GGC-GAC, Gly-Asp^), Hb J Bangkok (β^56GGC-GAC, Gly-Asp^), Hb Tak (β^146+AC, Ter-Thr^), Hb D Punjab (β^121GAA-CAA, Glu-Gln^), Hb S (β^6GAG-GTG, Glu-Val^), Hb Korle-Bu (β^73GAT-AAT, Asp-Asn^), Hb Malay (β^19AAC-AGC, Asn-Ser^), Hb C (β^6GAG-AAG, Glu-Lys^), Nakhon Ratchasima (α^63GCC-GTC, Ala-Val^), Hb Hekinan (α^27GAG-GAC, Glu-Asp^), Hb Beijing (α^16AAG-AAC, Lys-Asn^), Hb Lepore (δβ- hybrid Hb) and Hb A_2_-Melbourne [12]–[25]. Unknown Hb variants were further characterized by DNA sequencing.

### Identification of rare Hb variants previously un-described in Thailand

**Figure 2:**
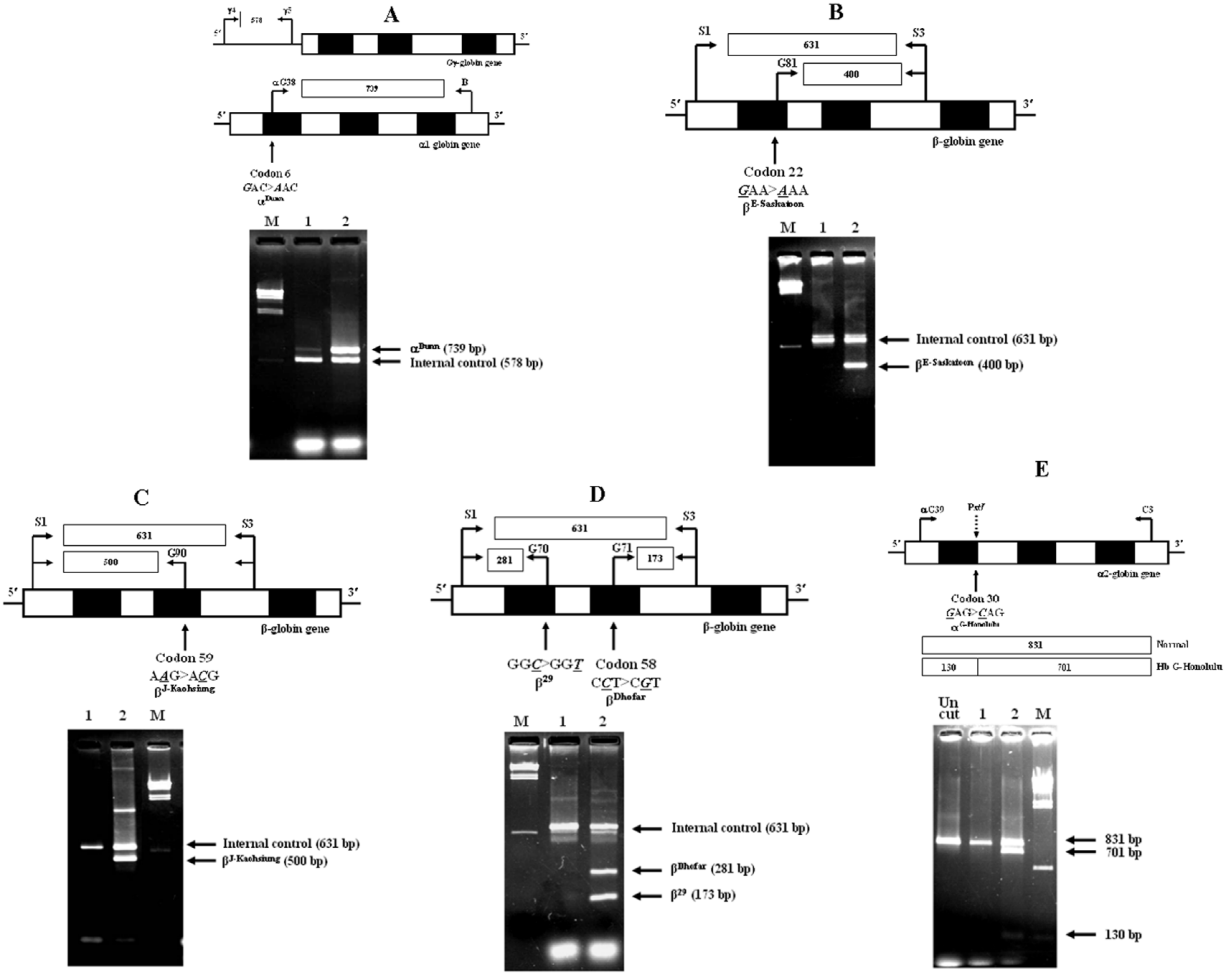
Molecular testing by allele specific PCR for detections of Hb Dunn (A), Hb E-Saskatoon (C), Hb J-Kaohsiung (D) and Hb Dhofar & β^29^ (E) and by PCR-RFLP assay for confirmation of Hb G-Honolulu (B). The locations and orientations of primers used in each assay are indicated. M represents the λ/*Hind*III size makers. In each representative gel electrophoresis; lane 1 and lane 2 are normal control and heterozygous for each corresponding Hb, respectively.

Allele-specific PCR (ASPCR) assays for identifications of Hb Dunn, Hb E-Saskatoon, Hb J-Kaohsiung and Hb Dhofar & β^29^ and the PCR-RFLP assay for detection of Hb G-Honolulu were developed as shown in **Figure 2** with the following details.

#### ASPCR for detection of Hb Dunn

The system was shown in **Figure 2A**. Primer αG38 (5′-ACCATGGTGCTGTCTCCTGCCA-3′) was used with primer B (5′-GAGGCCCAAGGGGCAAGAAGCAT-3′) to produce a specific fragment for α^Dunn^ mutation with 739 bp whereas primers γ4 (5′-GGCCTAAAACCACAGAGA-3′) & γ5 (5′-CCAGAAGCGAGTGTGTGGAA-3′) were used for internal control amplification. A total of 30 cycles after initial heating at 94°C for 3 min was performed under the following PCR condition: (94°C 1 min and 65°C 1 min 30 sec).

#### ASPCR for detection of Hb E-Saskatoon

This was shown in **Figure 2B**. With this system, primer G81 (5′-GGGCAAGGTGAACGTGGATA-3′) was used with common primer S3 (5′-TCCCATAGACTCACCCTGAA-3′) to produce specific fragment for β^E-Suskatoon^ mutation whereas primers S1 (5′-TGTCATCACTTAGACCTCAC-3′) & S3 were internal control amplification with the specific fragments of 400 and 631 bp, respectively. A total of 30 cycles after initial heating at 94°C for 3 min was performed under the following PCR condition: (94°C 45 sec, 60°C 1 min and 72°C for 45 sec).

#### ASPCR for detection of Hb J-Kaohsiung

As shown in **Figure 2C**, in this system, primers G90 (5′-CTTGCCATGAGCCTTCACCG-3′) and S1 and primers (S1 & S3) were used to produce the β^J-Ka^°^hsiung^ specific and internal control fragments with 500 bp and 631 bp, respectively. A total of 30 cycles after initial heating at 94°C for 3 min was performed under the following PCR condition: (94°C 45 sec, 65°C 1 min and 72°C for 45 sec).

#### Multiplex ASPCR for simultaneous detection of Hb Dhofar and β^29^ mutations

As Hb Dhofar and β^29^ mutations are located on the same β-globin gene, a simultaneous detection of both mutations is preferrable. We have therefore developed a multiplex PCR system as shown in **Figure 2D**. With this system, primer G70 (5′-GTAACCTTGATACCAACCTA-3′) specific primer was used with common primer S1 to generate the β^29^ mutation specific fragment whereas primers G71 (5′-GATGCTGTTATGGGCAACCG-3′) and S3 were used to produce specific fragment for the β^Dhofar^ mutation. Amplification from primers (S1&S3) could be used as internal control. Three specific fragments were respectively 173 bp, 281 bp and 631 bp in lengths, respectively. A total of 30 cycles after initial heating at 94°C for 3 min was performed under the following PCR condition: (94°C 45 sec, 65°C 1 min and 72°C for 45 sec).

The reaction mixture (50 µl) of the above mentioned PCR contained 50–200 ng genomic DNA, 30 pmoles of each primer, 200 µM dNTPs, 5% DMSO and 1 unit *Taq* DNA polymerase (New England Biolab, Inc., USA) in 10 mM Tris-HCl pH 8.3, 50 mM KCl, 1.5 mM MgCl_2_, 0.01% gelatin. All amplification reactions were carried out on a T-Personal Thermocycler (Biometra; GmbH, Gottingen, Germany).

#### PCR-RFLP assay for detection of Hb G-Honolulu

Since Hb G-Honolulu mutation creates a new restriction site for *Pst*I enzyme, on α2-globin gene, it could be detected by the PCR-RFLP assay as shown in Figure 2E. An α2 specific amplification was done using primers αG39 (5′-ACTCTTCTGGTCCCCACAGAC-3′) and C3 (5′-CCATTGTTGGCACATTCCGG-3′) and the amplified product was digested to completion with *Pst*I restriction enzyme (5′-CTGCA^▾^G-3′) (New England Biolabs, Beverly, MA, USA). The 831 bp Hb G-Honolulu derived fragment is digested into two fragments with 701 bp and 130 bp in lengths.

## Results

During 2002–2012, our referral laboratory at Khon Kaen University in northeast Thailand received 26,013 blood specimens for analysis of hemoglobinopathies from 24 provinces in different geographical areas throughout Thailand (**Figure 1**). Analysis using automated Hb analyzers and DNA assays revealed that 636 (2.4%) of them carried Hb variants, either alone or in combination with other hemoglobinopathies. A total of 32 different mutations were observed. Of these 636 subjects, 142 (22.4%) had α-globin chain variants with 13 different mutations, 451 (70.9%) had β-globin chain variants with 16 different types, 37 (5.8%) carried Hb Lepore (δβ-hybrid Hb) and the remaining 6 cases (0.9%) were carriers of δ-globin chain variant (**Table 1**).

**Table 1:** The frequency and Hb variant types found in 636 Thai referral cases throughout the countries.

Hb Variant (32 mutations)	Total no. (%)	Part of Thailand (n)				
		Center	Northeast	South	North	East
**α-Variant (13 mutations)**						
Hb Q-Thailand (α1^74GAC-CAC, Asp-His^)	**101 (15.8)**	55	40	5		1
Hb Siam (α1^15GGT-CGT, Gly-Arg^)	**8 (1.2)**	5	3			
Hb Queens (α1^34CTG-CGG, Leu-Arg^)	**6 (0.9)**	1		5		
Hb Beijing (α2^16AAG-AAC, Lys-Asn^)	**5 (0.8)**	1			4	
Hb Hekinan (α1^27GAG-GAC, Glu-Asp^)	**5 (0.8)**		5			
Hb Nakhonratchasima (α2^63GCC-GTC, Ala-Val^)	**5 (0.8)**	2	3			
Hb Dunn (α^6GAC-AAC, Asp - Asn^)	**3 (0.5)**		3			
Hb Thailand (α1^56AAG-ACG, Lys-Thr^)	**3 (0.5)**		3			
Hb St. Luke's-Thailand (α2 ^95CCG-CGG, Pro-Arg^)	**2 (0.3)**		2			
Hb Q-India (α1^64GAC-CAC, Asp-His^)	**1 (0.2)**		1			
Hb Phnom Penh (α1 ^117/118 insertion Ile^)	**1 (0.2)**		1			
Hb G-Honolulu (α2^30GAG-CAG, Glu-Gln^)	**1 (0.2)**	1				
HbJ-Wenchang-Wuming (α1 ^11AAG-CAG, Lys-Gln^)	**1 (0.2)**	1				
**β-Variant (16 mutations)**						
Hb Hope (β^136GGT-GAT, Gly-Asp^)	**121 (19.0)**	86	27	2	5	1
Hb Tak (β^146+AC, Ter-Thr^)	**90 (14.2)**	39	37	12	2	
Hb Malay (β^19AAC-AGC, Asn-Ser^)	**46 (7.2)**	9	10	27		
Hb J-Bangkok (β^56GGC-GAC, Gly-Asp^)	**43 (6.8)**	23	19	1		
Hb Pyrgos (β^83GGC-GAC, Gly-Asp^)	**42 (6.6)**	14	28			
Hb D-Panjab (β^121GAA-CAA, Glu-Gln^)	**38 (5.9)**	11	5	22		
Hb C (β^6GAG-AAG, Glu-Lys^)	**24 (3.8)**	8		16		
Hb Korle-Bu (β^73GAT-AAT, Asp-Asn^)	**19 (2.9)**	9	9	1		
Hb Cook (β^132AAA-ACA, Lys - Thr^)	**10 (1.6)**	2	8			
Hb Dhonburi (β^126GTG-GGG, Val-Gly^)	**8 (1.2)**		8			
Hb J-Kaohsiung (β^59AAG-ACG, Lys-Thr^)	**3 (0.5)**		3			
Hb Phimai (β^72AGT-ACT, Ser-Thr^)	**2 (0.3)**		2			
Hb Dhofar (β^58CCT-CGT, Pro-Arg^)	**2 (0.3)**	2				
Hb S (β^6GAG-GTG, Glu-Val^)	**1 (0.2)**			1		
Hb Raleigh (β^1GTG-GCG, Val-Ala^)	**1 (0.2)**		1			
Hb E-Saskatoon (β^22GAA-AAA, Glu-Lys^)	**1 (0.2)**		1			
**δβ hybrid Hemoglobin (2 mutations)**	**37 (5.8)**					
Hb Lepore-Hollandia (δ ^IVS I-42^/β^ IVS I-56^)		11	14			
Hb Lepore-Washington-Boston (δ^87^/β^ IVS II-8^)		10	2			
**δ variant (1 mutations)**	**6 (0.9)**					
Hb A_2_-Melbourne (δ^43GAG-AAG, Glu-Lys^)		4	2			
**Total (%)**	**636 (100)**	**294 (46.2)**	**237 (37.3)**	**92 (14.5)**	**11 (1.7)**	**2 (0.3)**

Among 13 α-globin chain variants observed, Hb Q-Thailand (α^74GAC-CAC, Asp-His^) was the most common one found in 101 cases (15.8%), followed by Hb Siam (α1 ^15GGT-CGT, Gly-Arg^) (n = 8; 1.2%) and Hb Queens (α1 ^34CTG-CGG, Leu-Arg^) (n = 6; 0.9%). Other less frequent variants included each of 5 cases (0.8%) with Hb Beijing (α2 ^16AAG-AAC, Lys-Asn^), Hb Hekinan (α1 ^27GAG-GAC, Glu-Asp^) and Hb Nakhon Ratchasima (α2 ^63GCC-GTC, Ala-Val^), each of 3 cases (0.5%) with Hb Dunn (α^6GAC-AAC, Asp-Asn^) and Hb Thailand (α^56AAG-ACG, Lys-Thr^), 2 cases (0.3%) of Hb St. Luke’s-Thailand (α2^ 95CCG-CGG, Pro-Arg^) and each one case (0.2%) of Hb Q-India (α1 ^64GAC-CAC, Asp-His^), Hb Phnom Penh (α1 ^117/118, Ile insertion^), Hb G-Honolulu (α^30GAG-CAG, Glu-Gln^) and Hb J-Wenchang-Wuming (α1 ^11AAG-CAG, Lys-Gln^).

For 16 different β-globin chain variants identified, Hb Hope (β^136GGT-GAT, Gly-Asp^) (n = 121; 19.0%) and Hb Tak (β^146+AC Ter-Thr^) (n = 90; 14.2%) were found to be the two most common ones. Other less common variants included Hb Malay (β^19AAC-AGC, Asn-Ser^) (n = 46; 7.2%), Hb J-Bangkok (β^56GGC-GAC, Gly-Asp^) (n = 43; 6.8%), Hb Pyrgos (β^83GGC-GAC, Gly-Asp^) (n = 42; 6.6%), Hb D-Panjab (β^121GAA-CAA, Glu-Gln^) (n = 38; 5.9%), Hb C (β^6GAG-AAG, Glu-Lys^) (n = 24; 3.8%), Hb Korle-Bu (β^73GAT-AAT, Asp-Asn^) (n = 19; 2.9%), Hb Cook (β^132AAA-ACA, Lys-Thr^) (n = 10; 1.6%) and Hb Dhonburi (β^126GTG-GGG, Val-Gly^) (n = 8; 1.2%). Other rare β-globin variants found with frequencies less than 1.0% included Hb J-Kaohsiung (β^59AAG-ACG, Lys-Thr^) (n = 3; 0.5%), Hb Phimai (β^72AGT-ACT, Ser-Thr^) (n = 2; 0.3%), Hb Dhofar (β^58CCT-CGT, Pro-Arg^) (n = 2; 0.3%) and each one case (0.2%) of Hb S (β^6GAG-GTG, Glu-Val^), Hb Raleigh (β^1GTG-GCG, Val-Ala^) and Hb E-Saskatoon (β^22GAA-AAA, Glu-Lys^).

In addition to these α- and β- globin chain variants, Hb Lepore, the hybrid δβ-globin chain namely Hb Lepore-Hollandia (δ^IVSI-42^/β^IVSI-56^) and Hb Lepore-Washington-Boston (δ^87^/β^IVSII-8^) were also detected in 37 cases (5.8%) and the remaining 6 cases (0.9%) were found to be carriers of the Hb A_2_-Melbourne, a δ-globin variant (δ^43GAG-AAG, Glu-Lys^).

**Figure 3:**
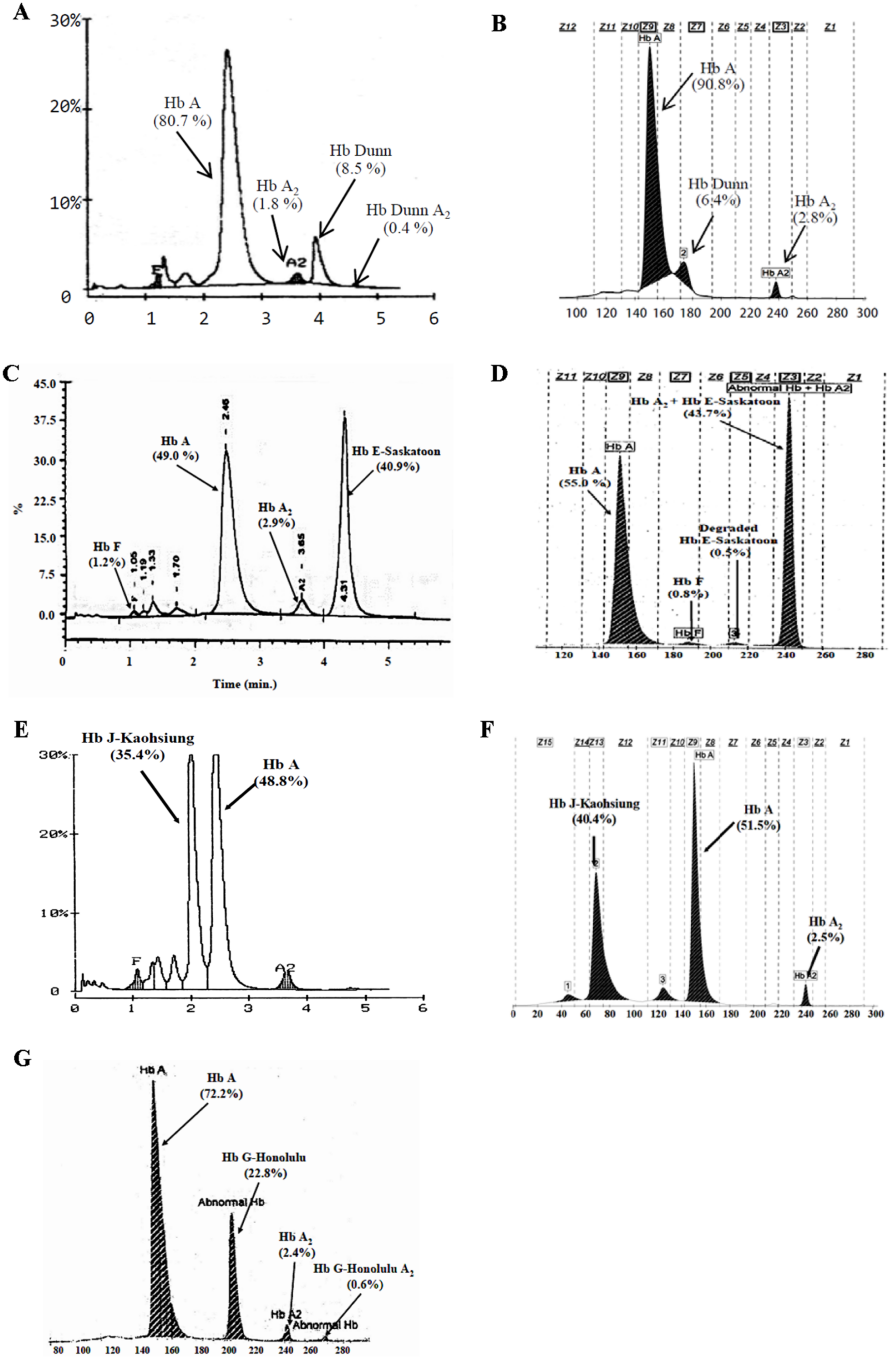
Separating profiles of the 4 rare Hb variants previously un-described in Thailand on HPLC and capillary electrophoresis, including Hb Dunn (A & B), Hb E-Saskatoon (C & D), Hb J-Kaohsiung (E & F) and Hb G-Honolulu (G).

**Figure 4:**
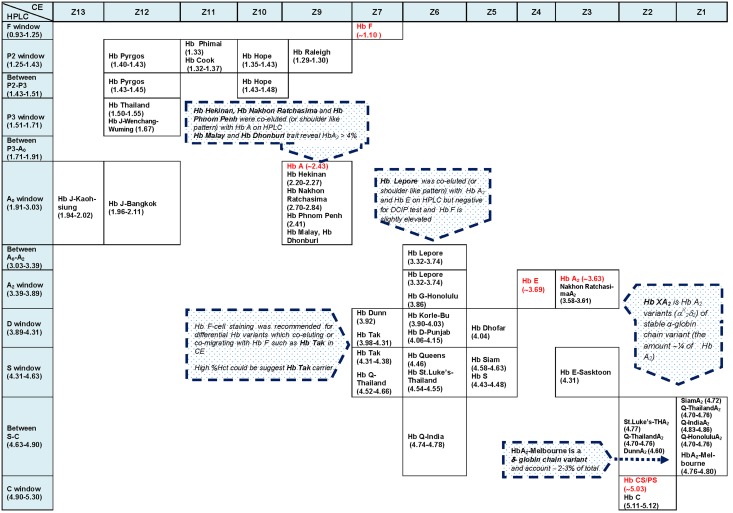
Retention times on HPLC and migration zones on CE of Hb variants found in Thailand, drawn perpendicular to allow two dimensional considerations for making presumptive diagnosis.

Among these 32 Hb variants, 7 variants were identified for the first time in Thai population, including Hb Dunn (α^6GAC-AAC, Asp-Asn^), Hb G-Honolulu (α^30GAG-CAG, Glu-Gln^), Hb E-Saskatoon (β^22GAA-AAA, Glu-Lys^), Hb J-Kaohsiung (β^59AAG-ACG, Lys-Thr^), Hb Phnom Penh (α1 ^117/118, Ile insertion^), Hb St. Luke’s-Thailand (α2 ^95CCG-CGG, Pro-Arg^) and Hb Raleigh (β^1GTG-GCG, Val-Ala^). The latter three Hb variants have been described in detail separately [26]–[28]. Hb separation profiles on HPLC and capillary electrophoresis of the remaining 4 Hb variants were presented in **Figure 3**. It is noteworthy that the numbers and amounts of abnormal Hbs observed could help in diagnostic consideration. Only one abnormal Hb peak (α_2_β^X^_2_) with the amount around 30–45% of total Hb would indicate a β-globin variant e.g. Hb E-Saskatoon (40.9%) and Hb Cook (40.8–45.8%). In contrast, α-globin variant is usually associated with more than one abnormal peaks i.e. variant of Hb A (α^x^_2_β_2_) with the amount around 25% in heterozygous state and that of Hb A_2_ (α^x^_2_δ_2_) e.g. in Hb G-Honolulu heterozygote in which 22.8% of Hb G-Honolulu and 0.2% Hb G-Honolulu-A_2_ were observed. The amount of δ- globin chain variant (α_2_δ^X^_2_) e.g. Hb A_2_-Melbourne herein detected is usually about half of the amount of Hb A_2_. This δ-globin chain variant is separated at Z1 on CE and near C-window on HPLC with the amount of 0.6–1.3%. Both Hb Lepore-Hollandia and Hb Lepore-Washington-Boston have the same separated behavior. They are co-eluted with Hb A_2_ and Hb E on HPLC but are separated at zone 6 of CE. Two other common Hb variants in our region, the Hb Constant Spring and Hb Paksé, are separated at the same migration zone with Hb C in Z2, as for other Hb A_2_ derivatives including Hb St. Luke’s-ThailandA_2_, Hb Q-ThailandA_2_ and Hb DunnA_2_. In contrast, Hb A_2_-variants of Hbs Siam, Thailand, Q-India, and G-Honolulu are displayed in Z1. Combined analysis using both HPLC and capillary electrophoresis is helpful. As shown in **Figure 4**, the retention times on HPLC of these Hb variants are drawn perpendicular with specific migration zones on capillary electrophoresis at the top of table, allowing two dimensional considerations. This could help in differentiation of these variants before being confirmed using molecular testing. For example, Hbs Pyrgos, Phimai & Cook, Hope and Raleigh are all eluted at the same P2 window on HPLC, they are clearly separated at zones 12, 11, 10 and 9 on CE, respectively. In contrast, while Hbs Pyrgos, Thailand and J-Bangkok are presented in zone 12 of CE, they are separated at different windows on HPLC. However, it is observed that some pairs of Hb variants e.g. (Hb Thailand & Hb J-Wenchang Wuming), (Hb Queens and Hb St. Luke’s-Thailand), (Hb Phimai & Hb Cook), and (Hb Korle-Bu and Hb D-Punjab) could not be clearly distinguished on both HPLC and CE systems, the result indicating a need for further characterization using molecular testing.

## Discussion

We have conducted for the first time a large scale survey and analysis of Hb variants in Thailand. The result indicates that Hb variants are very prevalence and heterogeneous in Thai population. Among 26,013 subjects investigated, 636 (2.4%) were found to carry Hb variants. As many as 13 α-Hb and 16 β-Hb variants in addition to a hybrid δβ Hb and a rare δ-globin chain variants were observed. It is conceivable that in area with high prevalence of thalassemia and hemoglobinopathies like Thailand [3]–[6], interaction between these globin gene defects resulting in complex hemoglobinopathies with complicated phenotypes and difficulty in laboratory diagnoses would be common. Interactions of some of these variants such as Hb Q-Thailand, Hb Hope, Hb Tak, Hb Korle-bu, Hb Beijing, Hb Hekinan, Hb J-Bangkok, Hb Pyrgos, Hb Siam, Hb Queens, Hb C, Hb D-Punjab, Hb Nakhon Ratchasima and Hb Phimai with thalassemia and hemoglobinopathies have been reported [12]–[30].

### α-Globin chain variants

For α-globin chain variants observed, Hb Q-Thailand is the most common one. This Hb variant is recognized on a chromosome with α^+^-thalassemia (4.2 kb deletion) and has been found to be caused by a single founder mutation in Thai population. Association of this Hb variant with α^0^-thalassemia leads to the α-thalassemia disease known as Hb QH disease [31]. We have described in details of other Hb variants including Hb Siam (α1 ^15GGT-CGT, Gly-Arg^), Hb Queens (α1 ^34CTG-CGG, Leu-Arg^), Hb Beijing (α2 ^16AAG-AAC, Lys-Asn^), Hb Hekinan (α1 ^27GAG-GAC, Glu-Asp^), Hb Thailand (α^56AAG-ACG, Lys-Thr^), Hb St. Luke’s-Thailand (α2 ^95CCG-CGG, Pro-Arg^), Hb Phnom Penh (α1 ^117/118, Ile insertion^) and Hb Nakhon Ratchasima (α2 ^63GCC-GTC, Ala-Val^) previously [13], [21]–[23], [26], [27]. The remaining Hb variants namely Hb Q-India (α1 ^64GAC-CAC, Asp-His^), Hb Dunn (α^6GAC-AAC, Asp-Asn^), Hb G-Honolulu (α^30GAG-CAG, Glu-Gln^) and Hb J-Wenchang-Wuming (α1 ^11AAG-CAG, Lys-Gln^), rarely been found in our population, are briefly described here.

#### Hb Q-India

Hb Q-India has been described in a Thai family in heterozygote form [32]. Here, we alternatively found Hb Q-India/Hb E syndrome caused by interaction of this Hb variant with Hb E. A Thai boy with this syndrome presented with mild hypochromic microcytosis with low MCV (64.0 fL) and MCH (22.0 pg), likely due to Hb E mutation. As for other α-globin chain variants, the relatively lower level of Hb E detected on capillary electrophoresis system could be due to the ability of the system to separate Hb E (α_2_β^E^_2_) (22.8%) from Hb Q-India-E (α^Q-India^_2_β^E^_2_) (3.1%) as well as Hb A_2_ (α_2_δ_2_) (2.8%) from Q-India-A_2_ (α^Q-India^_2_δ_2_) (0.2%) derivatives. The mutation could be detected by allele specific PCR assay shown in Figure 2.

#### Hb Dunn

Hb Dunn has been described in a black woman who had normal hematological features and no apparent clinical findings but has not been described in Thailand before. It has increased oxygen affinity and decreased stability [33]. The low levels of Hb Dunn in the 3 cases of our subjects (8.8–14.4% on HPLC & 6.4–6.9% on capillary electrophoresis) confirmed this. The Hb Dunn mutation could be detected by allele specific PCR assay shown in Figure 2.

#### Hb G-Honolulu

Hb G-Honolulu (also known as Hb G-Singapore, Hb G-Hong Kong and Hb G-Chinese) was first identified in a Chinese woman in Singapore [34], and was subsequently observed in several Chinese families but has not been found in Thailand. The mutation results in less negatively charged Hb molecule which causes Hb G-Honolulu eluted faster than Hb A on capillary electrophoresis system. In heterozygote form, both Hb G-Honolulu (α^G-Honolulu^_2_β^A^_2_) (22.8%) and Hb A_2_-G-Honolulu (α^G-Honolulu^_2_δ_2_) (0.2%) could be demonstrated on capillary electrophoresis. Carrier of this variant does not present the clinical symptom or significant hematological change. We demonstrated that since the Hb G-Honolulu mutation (α2; codon 30: GAG-CAG) creates a new restriction site for *Pst*I enzyme, it could be easily detected by a PCR-RFLP assay using *Pst*I (5′-CTGCAG-3′) digestion of the amplified α2-globin gene as shown in Figure 2.

#### Hb J-Wenchang-Wuming

Hb J-Wenchang-Wuming or Hb Anantharaj is a non pathological α- globin variant with more negative charge as compared to Hb A. It was first described in five members of a Chinese family of the Wuming district of China with relative amount of about 20% [35]. We found heterozygote form of this Hb variant in a Thai female with the level of 26% on HPLC and 25.3% in zone 12 of capillary electrophoresis, the amounts corresponding to a stable α1-globin variant. She had normal hematological feature. The separating profile of this variant is quite similar to Hb Pyrgos and Hb J-Bangkok [36], therefore diagnosis would preferably be performed by DNA testing.

### β-Globin chain variants

Regarding β-globin chain variants observed, Hb Hope (β^136GGT-GAT, Gly-Asp^) and Hb Tak (β^146+AC Ter-Thr^) are the two most common ones. While Hb Hope is clinically and hematologically innocuous, Hb Tak is a high O_2_ affinity variant. Diagnosis of Hb Hope by Hb analysis could be problematic as it co-migtrates with Hb F and may require Hb F test for differentiation [37]. In contrast, homozygosity for Hb Tak or compound heterozygosity for Hb Tak/δβ^0^-thalassemia have been documented with secondary erythrocytosis [38]. Other less common variants namely Hb Malay (β^19AAC-AGC, Asn-Ser^), Hb J-Bangkok (β^56GGC-GAC, Gly-Asp^), Hb Pyrgos (β^83GGC-GAC, Gly-Asp^), Hb D-Panjab (β^121GAA-CAA, Glu-Gln^), Hb C (β^6GAG-AAG, Glu-Lys^), Hb Korle-Bu (β^73GAT-AAT, Asp-Asn^), Hb Phimai (β^72AGT-ACT^) and Hb Raleigh (β^1GTG-GCG, Val-Ala^) have also been reported separately [14], [20], [28], [30], [36], [39]–[40]. Other less common ones are briefly described here.

#### Hb Cook

Hb Cook (β^132AAA-ACA, Lys-Thr^) with more negative charge as compared to Hb A has very similar HPLC and capillary electrophoretic profiles with those of Hb Hope and Hb Phimai. It was first reported in a child of Thai origin in combination with Hb E [41]. We found in this study both heterozygous Hb Cook and compound heterozygous for Hb Cook and Hb E. In pure heterozygote, the amounts of Hb Cook was found to vary between 40.8–45.8% on HPLC and 38.2–48.1% on capillary electrophoresis. In Hb Cook/Hb E syndrome, Hb A was absent and a higher level of Hb Cook as compared to Hb E was noted, the data indicating that a normal α-globin chain has more preferable to bind with β^Cook^ – globin chain than β^E^ – globin chain. Accurate diagnosis can be obtained on allele specific PCR assay shown in Figure 2.

#### Hb Dhonburi

Hb Dhonburi (β^126GTG-GGG, Val-Gly^) or Hb Neapolis has been thought to be a β^+^-thalassemia variant. Different origins of this abnormal Hb has been reported from Thailand, Italy and Iran [42]–[44]. Substitution of valine to glycine in the αβ contact region of the Hb molecule made it unstable and a positive in DCIP testing for Hb E. Since the Hb Dhonburi mutation does not change the net charge of Hb molecule, it is co-eluted and co-migrated with Hb A on HPLC and capillary eletrophoresis system. As expected for a β^+^-thalassemic hemoglobinopathies, we observed a borderline or slightly elevated Hb A_2_ level (3.5%–5.5%) and a mild hypochomic microcytosis in heterozygous subject.

#### Hb J-Kaohsiung

Hb J-Kaohsiung (β^59AAG-ACG, Lys-Thr^) is the fast β-globin chain variant with more negative charge than Hb A. It was first described in a Taiwanese male and later the same Hb variant namely the Hb J-Honolulu, was reported in members of a family of mixed Hawaiian-Chinese-Caucasian ancestry [45]. The mutation is at codon 59 where the normally occurring lysine is replaced by threonine. In this study, we observed the amounts of 43.8% in pure heterozygote and 40.4% in combination with α^+^-thalassemia. The amount decreased to approximately 35% when found in association with α^0^-thalassemia. This Hb variant appears to cause no anemia in the carrier. Accurate diagnosis can be obtained on allele specific PCR assay shown in Figure 2C.

#### Hb Dhofar

Hb Dhofar (β^58CCT-CGT, Pro-Arg^) is a Hb variant with more positive charge than Hb A. Hb Dhofar was first reported in Qara tribesmen living in Southern Arabia [46]. In addition to a mutation at codon 58 in exon 2 of β-globin gene, this Hb variant gene also carries another mutation in *cis* at codon 29 (β^29C-T^) in exon 1. The latter mutation creates an alternative splicing, leading to a β-thalassemia phenotype [47]. We also encountered two cases with heterozygous Hb Dhofar with low MCV (71.8 and 69.6 fL) and MCH (23.0 and 22.6 pg) values. The level of Hb Dhofar detected were as expected around 20% on HPLC and Hb A_2_ was detected between 3.5–3.9% on capillary electrophoresis; indicating a β^+^-thalassemia nature of this variant. We observed both mutations in our subjects which could be detected simultaneously by a multiplex allele PCR assay as shown in Figure 2D.

#### Hb E-Saskatoon

Hb E-Saskatoon (β^22GAA-AAA, Glu-Lys^) was found in several families of Scottish descent, Turkish, Spainish, and Japanese [48] but has not been reported in Thai population. We found this variant in a healthy man who carried an abnormal Hb at the S window on HPLC (the amount of which was 40.9%). This Hb variant co-migrated with Hb A_2_ in zone 3 of capillary electrophoresis with the amount of 44.2%. Hb E-Saskatoon does not cause any major hematological problem in homozygous or in compound heterozygous state with β-thalassemia [49]–[50]. As for other variants, it could be detected by allele specific PCR as shown in Figure 2B.

It is conceivable from this study that with heterogeneity of the Hb variants found and resolution limitation of each Hb analyzer, combined analysis using both HPLC and capillary electrophoresis is preferable where possible for making presumptive diagnosis of the cases using two dimensional interpretations as shown in **Figure 4**.

## Conclusion and Implication

This study reported in a large cohort of Thai population, the prevalence, frequency and heterogeneity of abnormal Hbs found in Thailand and the diagnostic consideration for routine setting. Although most Hb variants have minimal clinical significance, differential diagnosis from other clinically relevant variants is essential. Because of high prevalence of thalassemia in the population, interaction of different forms of Hb variants and thalassemia causing complex syndromes with difficulty in laboratory diagnosis would be common in Thai population. Where possible combined analysis of Hb using different methods e.g. HPLC and capillary electrophoresis would help in initial recognition and providing presumptive diagnosis of the cases before being confirmed by appropriate DNA assays. The ethnic background of the patients, complete hematological and Hb analysis data as well as the nature of corresponding mutations of the variants should also prove useful for further population genetic study of human Hb variants in the population.
